# A Review of Artificial Intelligence Applications in Hematology Management: Current Practices and Future Prospects

**DOI:** 10.2196/36490

**Published:** 2022-07-12

**Authors:** Yousra El Alaoui, Adel Elomri, Marwa Qaraqe, Regina Padmanabhan, Ruba Yasin Taha, Halima El Omri, Abdelfatteh EL Omri, Omar Aboumarzouk

**Affiliations:** 1 College of Science and Engineering Hamad Bin Khalifa University Doha Qatar; 2 National Center for Cancer Care and Research Hamad Medical Corporation Doha Qatar; 3 Surgical Research Section Department of Surgery Hamad Medical Corporation Doha Qatar; 4 College of Medicine Qatar University Doha Qatar; 5 College of Medicine University of Glasgow Glasgow United Kingdom

**Keywords:** cancer, oncology, hematology, machine learning, deep learning, artificial intelligence, prediction, malignancy, management

## Abstract

**Background:**

Machine learning (ML) and deep learning (DL) methods have recently garnered a great deal of attention in the field of cancer research by making a noticeable contribution to the growth of predictive medicine and modern oncological practices. Considerable focus has been particularly directed toward hematologic malignancies because of the complexity in detecting early symptoms. Many patients with blood cancer do not get properly diagnosed until their cancer has reached an advanced stage with limited treatment prospects. Hence, the state-of-the-art revolves around the latest artificial intelligence (AI) applications in hematology management.

**Objective:**

This comprehensive review provides an in-depth analysis of the current AI practices in the field of hematology. Our objective is to explore the ML and DL applications in blood cancer research, with a special focus on the type of hematologic malignancies and the patient’s cancer stage to determine future research directions in blood cancer.

**Methods:**

We searched a set of recognized databases (Scopus, Springer, and Web of Science) using a selected number of keywords. We included studies written in English and published between 2015 and 2021. For each study, we identified the ML and DL techniques used and highlighted the performance of each model.

**Results:**

Using the aforementioned inclusion criteria, the search resulted in 567 papers, of which 144 were selected for review.

**Conclusions:**

The current literature suggests that the application of AI in the field of hematology has generated impressive results in the screening, diagnosis, and treatment stages. Nevertheless, optimizing the patient’s pathway to treatment requires a prior prediction of the malignancy based on the patient’s symptoms or blood records, which is an area that has still not been properly investigated.

## Introduction

### Background on Hematologic Malignancies

Blood cancers, namely leukemia and lymphoma, are generally ranked among the most common and deadliest cancer types [1]. Typically, hematologic malignancies result from abnormal growth of white blood cells (WBCs) in the human body, which creates a disproportion among blood elements [2]. The blood contains red blood cells (RBCs), WBCs, and platelets. The role of RBCs is to transmit oxygen from the heart to the entire system, and they constitute the largest proportion of the blood volume [3]. By contrast, WBCs play a crucial role in protecting the body from diseases and infection by deploying various immune mechanisms [4]. Hence, maintaining a healthy WBC level is imperative for the protection of the human body. Normally, these blood elements mature and replenish depending on the body’s needs [5]. However, their growth can become disordered when certain hematologic malignancies are present [6]. For instance, because of the considerable increase in the number of abnormal WBCs, the ability of bone marrow to generate and support healthy RBCs and platelets in terms of oxygen and nutrition supply is impaired [2,7]. Moreover, these malignant WBCs can circulate throughout the body via blood and cause irreparable damage to other organs such as the liver, kidney, and brain [8].

### Challenges in Hematology Management

Although complete blood count (CBC) tests often serve as the first step in detecting hematologic malignancies by identifying abnormal blood cell count or any distortion in cell morphology, this simple test is generally deemed insufficient for a practitioner to diagnose blood cancer [5,9]. Therefore, several microscopic evaluations of the blood smear are performed to reach a final diagnosis [5]. As all the available methods are manual and require highly skilled medical personnel for interpretation, a blood cancer diagnosis can be costly and time consuming, which negatively impacts the patient’s efficient and timely treatment [10]. Another challenging aspect in hematology detection is that WBCs are surrounded by other blood components. Thus, the current identification method of manually counting the number of WBCs that appear abnormal does not provide accurate classification results [11]. In fact, it has been reported that diagnostic delays mainly occur because of the complexity of symptom analysis and challenges associated with disease diagnosis.

### Artificial Intelligence in Hematology Management

Motivated by the remarkable achievements of artificial intelligence (AI) in various fields, the applicability of such algorithms in solving critical problems related to oncology and hematology was recently investigated and proven efficient [6]. In particular, machine learning (ML) and deep learning (DL) methods were used to assist to classify various cancer types, facilitate faster diagnosis, and provide a basis for accurate clinical decisions for better health outcomes. The 2 main challenges in the implementation of AI in medicine are the limitation and restriction of health information.

This review provides a comprehensive contribution to the hematology care management field, with the objective of studying the applications of AI in various blood cancer stages and detecting the limitations of ML- and DL-based models that have been previously implemented. This paper attempts to highlight the existing gaps in the field of hematology management by studying, classifying, and analyzing 144 papers published between 2015 and 2021.

The “Methods” section clarifies the methodology used to perform the search of all reviewed papers. The “Results” section discusses and classifies existing research based on malignancy type and stage, respectively. Finally, the “Discussion” section provides a detailed analysis of AI applications for hematologic malignancies.

## Methods

**Figure 1:**
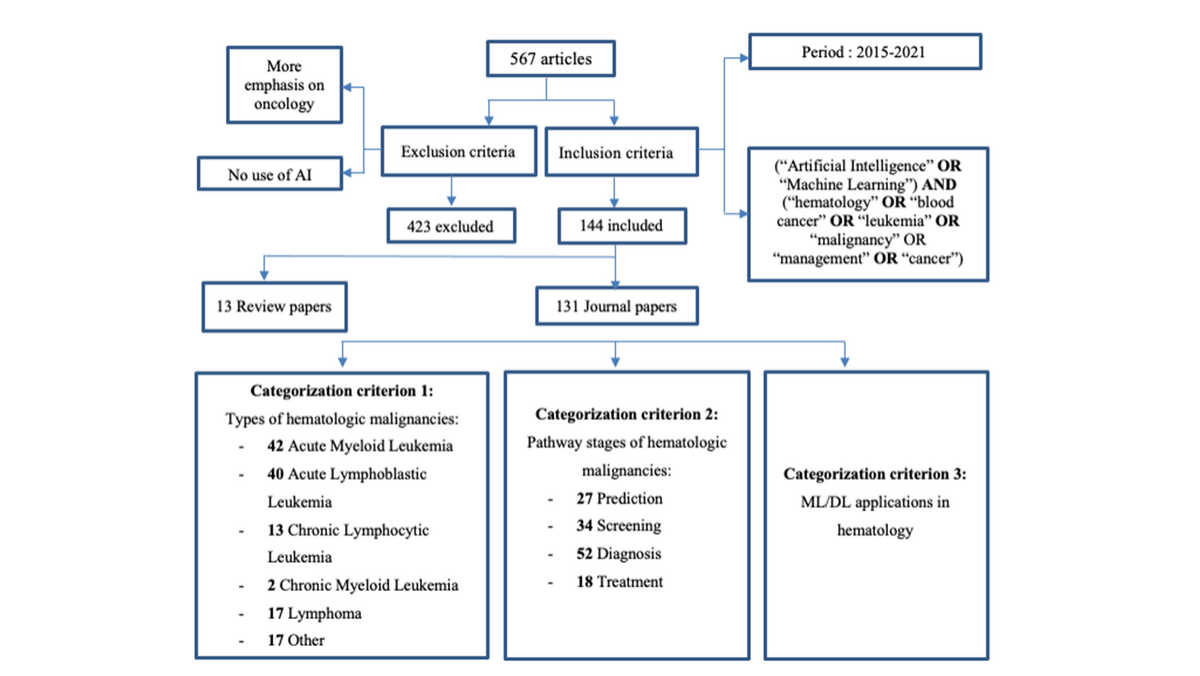
Review chart showing paper elimination and categorization process. AI: artificial intelligence; DL: deep learning; ML: machine learning.

In this review, peer-reviewed and publicly available journal papers were identified from a variety of online databases, while publicly unavailable copies were obtained through our institutional access to journal publications and databases. The search for appropriate journal papers was performed using specific keywords such as AI, ML, blood cancer, hematology, malignancy, leukemia, management, and cancer, which were linked and combined using 2 Boolean operators “AND” and “OR” to produce more focused outcomes (Figure 1).

The paper collection process targeted all the articles written in English in the last 7 years with the aforementioned keywords in their abstracts or titles. The search identified 567 papers, of which only 144 were retained for review. The 423 excluded papers either focused on oncology without paying special attention to hematology, addressed hematologic malignancies from an automated perspective without employing AI models, or covered purely technical drug treatments for blood cancers.

## Results

### Overview

This section presents the paper collection results, as well as an analysis of the literature and its classification with respect to the study category, malignancy type, and pathway stage.

**Figure 2:**
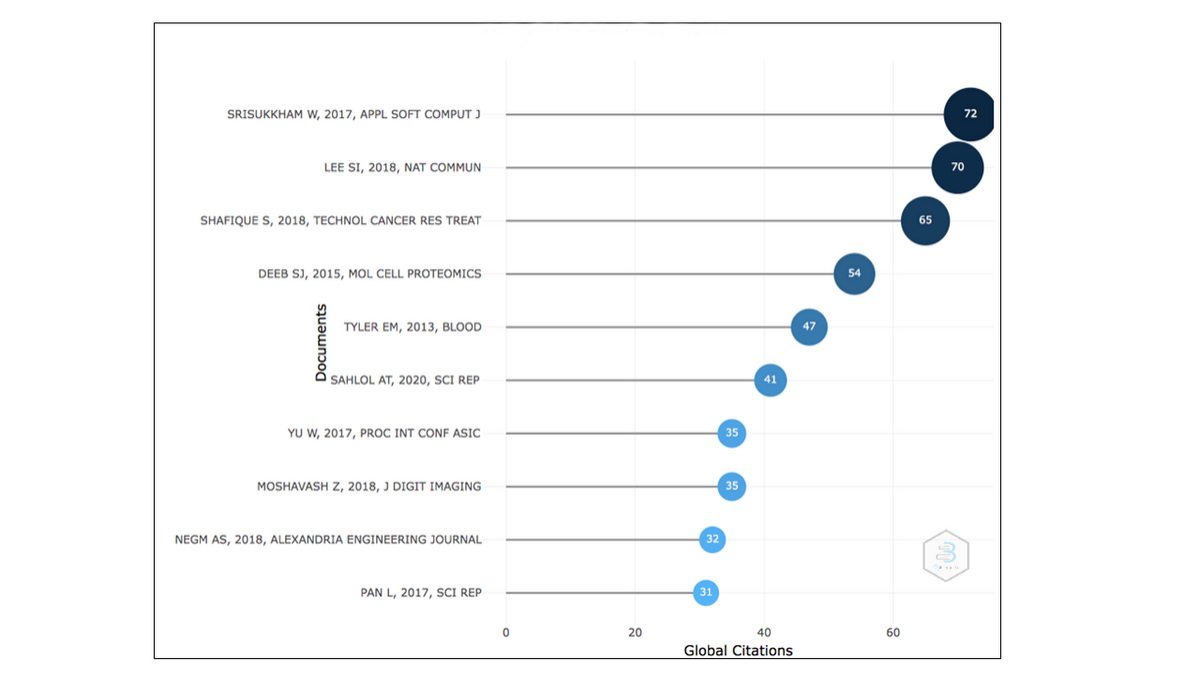
Citation statistics for the most cited documents.

From the analysis of the selected papers, Figure 2 lists the top 10 most globally cited documents in the field of AI in hematology management, with “Intelligent leukemia diagnosis with Bare-bones PSO based feature optimization” [12] rated as the most cited article with 72 citations globally.

### Descriptive Analysis

**Figure 3:**
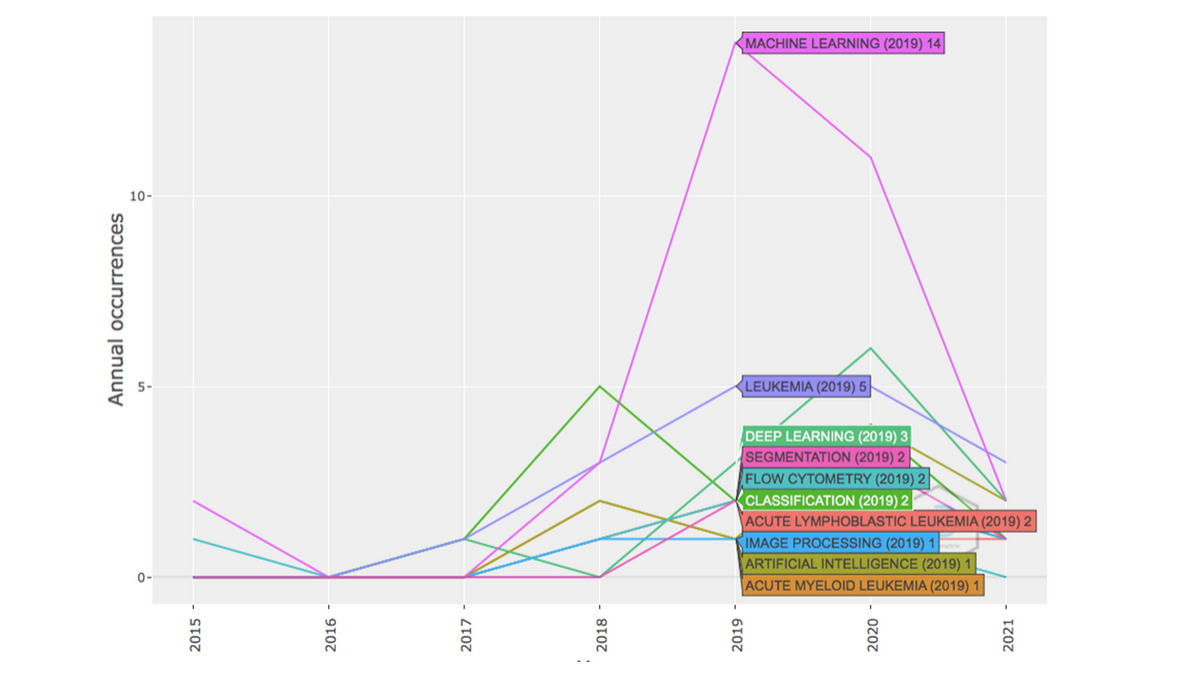
Thematic evolution of hematology management research.

With the emergence of AI and the remarkable results it has achieved over the last decade in many areas, researchers in the field have recently started to investigate its applications in blood cancer management. Figure 3 maps the evolution of thematic research in the field of hematology. We can clearly see a spike in the year 2019 in the occurrence of ML and leukemia, followed by DL, AI, and classification trends in the year 2020, which indicates the progression and development of ML and DL themes and their applications in the blood cancer field as well as the recent focus on AI to drive hematologic research.

Furthermore, keywords are used to outline the content of different articles. An analysis of the high-frequency keywords can give an idea about the current research status and potential future directions in the area of AI in hematology management.

**Figure 4:**
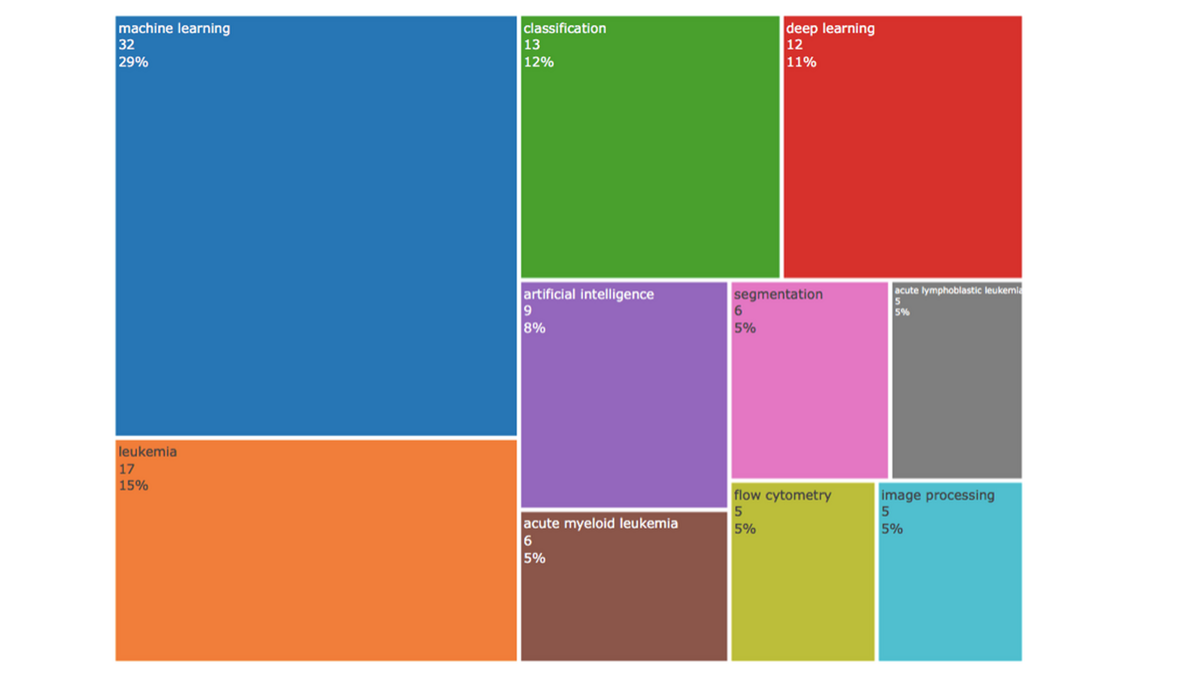
Statistics for most cited keywords.

Using the mapping and analysis tools provided in the Biblioshiny software (K-Synth Srl), a word tree map was generated to highlight the top 10 most frequently used keywords by scholars in the field. Figure 4 shows the hierarchical visualization of author keywords.

The highest frequencies were for ML (29%), leukemia (15%), classification (12%), and DL (11%), with 32, 17, 13, and 12 occurrences respectively, indicating the recent focus on ML and DL applications in the detection and classification of leukemia and its types (acute myeloid leukemia [AML] and acute lymphoblastic leukemia [ALL]). The tree map also highlights the different techniques and tools used in this field of research through keywords such as “segmentation,” “image processing,” and “flow cytometry,” with an identical frequency of 5% each.

### Category Selection

In the process of analyzing and classifying the set of collected articles, the generated documents were categorized based on the blood malignancy type, pathway stage, and ML/DL techniques used. The collected papers were either reviews of the literature, journal papers, or conference proceedings. Nonetheless, special attention was paid to review papers, as they provided a clear idea of the research that has been carried out as well as the gaps that remain to be addressed. Table 1 provides a detailed classification of the collected articles by category.

**Table 1:** Classification of journal papers by category.

Categories	Number of articles	Studies
Review papers	13	[5,13-24]
Conference proceedings	33	[1,4,6,10,11,25-52]
Journal articles	98	[2,3,7-9,12,53-144]

### Material Evaluation: Hematologic Malignancy Types

After excluding the reviews, this section classifies the 131 remaining journal papers based on the malignancy type and pathway stage.

Although leukemia and lymphoma share some common symptoms, there are major differences in their origins, causes, and treatments. Leukemia is a slowly developing disease that can be either chronic or acute [12]. Acute leukemia spreads quickly, whereas chronic leukemia develops more slowly during its initial stages and is generally more common [33]. For both types, leukemia can be myeloid, which affects the myeloid cells that give rise to WBCs; or lymphoblastic, which starts in cells that later become lymphocytes. Lymphoma, by contrast, affects the lymph nodes. In the case of lymphoma, WBCs (B and T lymphocytes) exhibit abnormal proliferation and based on the presence or absence of Reed-Sternberg cells, they are classified as either Hodgkin or non-Hodgkin diseases, respectively. Table 2 classifies the studies based on the type of malignancies that they address, as either acute myeloid/lymphoblastic leukemia, chronic myeloid/lymphoblastic leukemia, lymphoma, or other less common types of hematologic diseases.

Among the aforementioned malignancy types, AML is the most addressed hematologic disease in the literature with an occurrence of 32.1% (42/131) in the collected journal papers, followed by ALL. Apparently, the applicability of AI methods in managing chronic lymphocytic leukemia (CLL; 13/131, 9.9%) and chronic myeloid leukemia (CML; 2/131, 1.5%) are the least explored areas, wherein further research is pivotal.

**Table 2:** Distribution of the 131 studies based on malignancy type.

Malignancy type	Values, n (%)
Acute myeloid leukemia	42 (32.1)
Acute lymphoblastic leukemia	40 (30.5)
Chronic lymphocytic leukemia	13 (9.9)
Chronic myeloid leukemia	2 (1.5)
Lymphoma	17 (13.0)
Other	17 (13.0)

### Pathway Stages of Hematologic Malignancies

#### Prediction

The absence of symptoms during the early stages of leukemia makes it challenging for practitioners to predict the occurrence of cancer [117]. Typically, leukemia detection relies on the use of blood cell image classification methods [29]. The introduction of AI-based models is intended to enhance identification accuracy and provide early prediction of potential spread within the human body to help increase the chances of patient recovery and survival.

#### Screening

No standardized leukemia screening tests have been proven reliable and efficient enough to identify the presence of blood cancer in its early stages [83]. The causes of leukemia remain unknown and a diagnosis cannot be confirmed until the initial symptoms develop. Thus, there is ongoing research on imaging techniques, screening methods, and ways to increase the contribution of AI to provide a better understanding of the root causes of this condition and thus enable expert hematologists to identify and diagnose blood malignancies accurately [56].

#### Diagnosis

For many diseases, diagnosis is the most crucial stage in the illness pathway [85]. As the field of hematologic research is expanding in an unprecedented manner, the emergence of AI provides an opportunity to overcome the challenge of handling large amounts of imaging data, improve the efficiency and quality of hematologic pattern identification, and provide a clear understanding of suitable therapies following a precise diagnosis [87].

#### Treatment

AI integration into blood cancer treatment has enabled many advanced practices to deliver timely and efficient therapy [20]. Several techniques were used to identify the doses and combinations of drugs required for each patient as functions of their health status, age, and other essential factors. Table 3 classifies the 131 journal papers based on their corresponding pathway stage.

While there has been more emphasis in the literature on the diagnosis stage, which represents 39.7% (52/131) of the total journal papers collected, treatment and prediction are the least investigated pathway stages with only 13.7% (18/131) and 20.6% (27/131), respectively. This is mainly due to the difficulty in identifying crucial biomarkers that can be used as discriminative feature variables to predict the disease before the onset of symptoms. Similarly, in the case of treatment, as detailed in later sections of this paper, most of the AI-based models [9,42,75] reported in the treatment phase utilize gene expression profiles for model development, which are relatively complex. Hence, more research is needed to investigate the feasibility of easily available biological features for the prediction of treatment-related disease relapse and patient survival.

**Table 3:** Classification of journal publications by pathway stage (N=131).

Pathway stage	Values, n (%)	Studies
Prediction	27 (20.6)	[1,6,7,29,38,40,41,50,51,53,55,64,69,82,92,94,96,99-102,105,117,118,136,143,144]
Screening	34 (26.0)	[3,10,28,30,32-34,36,44-46,57,58,61,66,71,72,76-78,80,83,85,89,91,106,112,115,119,124,129,130,132,138]
Diagnosis	52 (39.7)	[2,4,8,11,12,25-27,31,35-37,39,43,47-49,52,54,60,62,63,65,67,68,70,79,84,86-88,90,95,97,98,107,108, 110,111,113,116,120-123,125-128,133,134,137]
Treatment	18 (13.7)	[9,42,56,59,73-75,81,93,103,104,109,114,131,135,139-141]

## Discussion

This section analyses the existing literature with respect to the methods applied (ML or DL) for solving issues in each pathway stage.

### Hematology Prediction

#### Machine Learning–Based Models

Childhood ALL is a malignant cancer that is the leading cause of pediatric cancer mortality, and around 20% of the children fully treated end up having a recurrence [131]. Hence, it is crucial to predict relapse to deal with the multiple risk groups accordingly. For better management and follow-up planning, Pan et al [131] introduced an ALL relapse prediction model based on ML algorithms that help classify patients with ALL into their appropriate risk categories. In the model selection process, 103 clinical variables were used to train 4 classification algorithms, random forest (RF), decision tree, support vector machine (SVM), and linear regression, to distinguish relapses from nonrelapses in the 3 clinically predefined risk categories: standard-, intermediate-, and high-risk levels. While Pan et al [131] built a model to predict disease relapse, Hauser et al [144] studied the possibility of predicting CML prior to diagnosis using only CBC test results and ML algorithms such as XGBoost and LASSO algorithms on 1623 patients with a definitive CML status. The variables used in the study included laboratory CBC test results, patient demographic features such as their age and gender, and patient encounter information (the number of patient visits to outpatient clinics, etc.). A forward feature selection process was employed to measure the predictive performance of the most potential predictors. The data set was then divided into 7 subsets, in which time of diagnosis was set as a patient baseline, and the 6 remaining sets corresponded to the different time periods preceding the diagnosis test. Interestingly, variable selection yielded different features for inclusion in the models depending on the data collection interval.

The performances of the chosen classifiers in [131] were evaluated by a 10-fold cross validation in each of the 100 training sets. However, the suggested approach is considered insufficient for internal validation that requires at least 50 repeats [145]. By contrast, the chosen data set in [144] was divided into 2 distinct groups: train/validation and test groups. While the latter split-sample validation approach seems reasonable and justifiable to use in this case given the large sample size, potential drawbacks may arise, and several aspects still need attention throughout the application. For instance, as the sample split was performed fully at random, substantial patient imbalances might have occurred with respect to the distributions of predictors and the output. Moreover, 20% was used for model assessment leading to a potential biased evaluation of the model’s performance [145].

Furthermore, it is well known that patients with leukemia often suffer from health issues due to frequent infections, which can lead to death if it is not detected early. Toward this end, Agius et al [55] investigated the risk of infection due to a weakness in the immune system or a cytotoxic treatment immediately after CLL diagnosis by developing the CLL Treatment-Infection Model (CLL-TIM). For each patient, the prediction point was set at 3 months after their diagnosis, and the target output was the 2-year infection risk or CLL treatment. After excluding 74 patients who died and 373 who initiated their treatment before the prediction point, the study cohort’s final size corresponded to 3729 patients. In contrast to Hauser et al [144], Agius et al [55] employed stratified sampling to maintain class distributions and compensate for the 52% International Prognostic Index for CLL (CLL-IPI) missing variables, by dividing the data set into 65% training set and 17.5% for each of the test and internal validation sets. Using 7288 features resulting from a collection of variables from different sources, comprising baseline variables at the time of diagnosis including age, gender, etc.; routine laboratory tests; microbiology findings; pathology reports; and diagnosis codes for all patients, the CLL-TIM ensemble algorithm was composed of 28 ML models that could identify patients at a high risk of infection to increase their chances of survival. The model was then validated on both internal and independent external test cohorts, and exhibited interesting performances, surpassing the CLL-IPI.

#### Deep Learning–Based Models

Hassouneh et al [50] suggested the use of deep neural networks (DNNs) to predict survivability of patients with leukemia to boost the psychological state of patients and enable physicians to arrange the proper treatments for different cases. The final DNN structure encompassed 6 hidden layers, with 45 hidden neurons in each corresponding hidden layer, and a dropout activation with 25%. While Hassouneh et al [50] used patient records and attributes of patients with leukemia for modeling, Boldú et al [134] relied on a set of 731 blood smear images to predict initial patient diagnosis. For data set limitation purposes, 4 convolutional neural network (CNN) models were pretrained on a larger data set and used for the aforementioned task. Their respective performances were compared and the architecture achieving the best outcome was trained further. Next, these pretrained CNNs were fine-tuned to match the type of data that are fed into the model. Finally, the proposed ALNet model was able to distinguish, on a first level, between healthy and abnormal blood cell images. On a second level, it was able to identify whether the blasts were myeloid or lymphoid. After a thorough evaluation process using 5-fold cross validation and the hold-out (80%/20%) approach with 470 iterations, ALNet demonstrated interesting results and was chosen as the best architecture for modeling. One strength of this study is that the 5-fold cross validations were approximately balanced, and despite the random split, the data from the same patient smear were maintained within the same fold. Alternatively, the proposed DNN algorithm in [50] was trained and evaluated using 2 methods: 10-fold cross validation and ensemble method. Although the 10-fold cross-validation method is known to generate more stable results, the number of repetitions is quite crucial, which was absent in this study [145].

Overall, it is very clear that all the aforementioned algorithms succeeded in predicting the different aspects and repercussions of the disease. Nevertheless, no existing literature has yet examined the root cause of the malignancy by predicting the possibility of patient infection. Table 4 summarizes some studies in the prediction phase alongside their objectives, the data sets used in the studies, the methodologies followed, the performance of the models applied, their strengths and weaknesses, and the validation approach used, where available.

**Table 4:** Study analysis for journal publications on the prediction phase.

Reference	Objective, data set, and methodology	Performance and remarks
[131]	Objective: ALL^a^ relapse prediction Data set: 336 newly diagnosed children with ALL Methodology: Random forest algorithm	Performance: Accuracy: 0.829 AUC^b^: 0.902 Strengths: Usage of 4 ML^c^ algorithms and 104 features Good model performance in all risk-level groups Adoption of a special feature selection strategy: 100-fold Monte Carlo cross validation combined with 10-fold cross validation Limitations: Data set imbalance (relapsed and nonrelapsed children) Strong predictors were excluded from the variable set Validation: 10-fold cross validation
[144]	Objective: Prediction of patients with CML^d^ and non-CML using complete blood count records Data set: Complete blood count records of 1623 patients with a BCR-ABL1 test extracted from the US Veterans Health Administration Methodology: XGBoost and LASSO	Performance: AUC range: 0.87-0.96 at the time of diagnosis Strengths: Use of 2 models Use of 2 feature selection methods Limitations: Imbalanced data set (predominant gender is male) Nonstandard data collection process Validation: Split sample validation (20% of the data for validation)
[1]	Objective: Leukemia detection based on biomedical data Data set: 401 leukemia datapoints from Z H Sikder Medical College and Hospital Methodology: Decision tree	Performance: Accuracy: 100% Strengths: Use of 4 supervised ML algorithms Limitations: Overfitting Validation: 10-fold cross validation
[50]	Objective: Prediction of leukemia survivability Data set: 131,615 records and 133 attributes for patients with leukemia from the SEER^e^ database Methodology: Deep neural network model	Performance: Accuracy: 74.85% Strengths: Use of a DNN^f^ ensemble method Limitations: Many problems in the leukemia data set (redundant attributes, missing values, and unknown values) Validation: 10-fold cross validation Ensemble method
[96]	Objective: Predictive identification of patients at risk during treatment Data set: 737 samples of patients diagnosed with CLL^g^ at Mayo Clinic Methodology: logistic regression, support vector machine, gradient boosting machine, random forest	Performance: ROC^h^-AUC: above 80% Strengths: Binary classification outperforms survival analytic methods Limitations: Lack of actionable information provided by the ML algorithms Validation: 100 runs of 5-fold cross validation

^a^ALL: acute lymphoblastic leukemia.

^b^AUC: area under the curve.

^c^ML: machine learning.

^d^CML: chronic myeloid leukemia.

^e^SEER: Surveillance, Epidemiology, and End Results

^f^DNN: deep neural network.

^g^CLL: chronic lymphocytic leukemia.

^h^ROC: receiver operating characteristic

### Hematology Screening

#### Machine Learning–Based Models

Initial leukemia screening and its efficient diagnosis require a deep and thorough image analysis process. As opposed to traditional manual screening, automated leukemia screening is a novel approach that minimizes human interaction and provides more accurate clinical information by using blood smear images to identify ALL automatically [17]. The automated screening process is considered challenging due to the leukocyte localization and region extraction phases, which are generally obtained via background removal and separation of surrounding blood components that might distort the overall detection process. For this reason, many studies have employed techniques such as principal component analysis as a filter to identify any features that do not bring any important information to the classification process [115] to enhance detection accuracy. Similarly, Chebouba et al [32] used a meta-heuristic stochastic local search technique to select the most important genes and proteins to be used in the RF-based classification of patients with AML.

#### Deep Learning–Based Models

CML consists of 3 sequential phases that change based on the patient’s status and can progress to more severe phases if timely treatment is not provided. This makes CML phase identification very crucial, as different phases require separate treatments and medical regimens. In the chronic phase, less than 10% of the cells in both the blood and bone marrow are blasted. The severity and persistence of the aforementioned phase depend mainly on the consistency of the therapy followed. If the chronic stage is neglected and the patient does not receive timely and effective treatment, the condition can deteriorate to reach an accelerated phase where the blast count increases to around 10%-19%. Similarly, if the patient’s condition declines with no appropriate medical intervention, the percentage of blasted WBCs doubles to reach around 20% or more. At this stage, the patient’s state is considered uncontrollable, and the patient starts to exhibit symptoms such as fever, weakness, and weight loss [33]. As CNNs were proven to be efficient tools for accurate image recognition, Khosla and Ramesh [33] suggested using the latter to classify different CML images into their respective phases. Similarly, Togacar et al [71] used CNNs to separate WBC images into their 4 subclasses: eosinophil, lymphocyte, monocyte, and neutrophil. While the aforementioned CNNs are successful in identifying the most important features in images with no human supervision, they generally require large training data to achieve high performance, and their employment is regarded expensive in terms of both time and training. For this reason, while many studies tend to employ image augmentation to the existing data set to create larger samples by slightly changing the existing collected images [33], others implement the concept of transfer learning to overcome the training data shortage. The idea behind the latter is to leverage the power and knowledge of a pretrained model to apply it on a new similar task. For instance, Sahlol et al [3] proposed a novel approach consisting of a hybrid model and combined CNN feature extraction using the solid architecture of VGGNet that was pretrained on ImageNet, to separate malignant cells from benign ones. Similarly, Li et al [127] developed the globally optimized transfer deep-learning platform with multiple pretrained CNNs (GOTDP-MP-CNNs). This DL platform is composed of 17 CNNs able to classify pathologic images into human diffuse large B-cell lymphoma and non–diffuse large B-cell lymphoma. Table 5 summarizes some of the collected studies in the screening phase.

**Table 5:** Study analysis for journal papers in the screening phase.

Reference	Objective, data set, and methodology	Performance and remarks
[3]	Objective: Classification of white blood cell leukemia Data set: Acute Lymphoblastic Leukemia Image Database for Image Processing 1 and 2 Methodology: A hybrid model (CNN^a^ and SESSA^b^)	Performance: Accuracy: 99.2% Sensitivity: 100% Strengths: Powerful performance using CNN Use of the salp swarm optimization method Hybrid classification method Use of transfer learning Limitations: Small limited data set insufficient to train CNNs Validation: 5-fold internal cross validation and 20% testing (external validation)
[61]	Objective: Automated identification of acute lymphoblastic leukemia Data set: Blood smear images obtained from the Department of Hematology at the University Hospital Ostrava Methodology: support vector machine/artificial neural networks	Performance: Accuracy: 98.19% Strengths: High classification accuracy Successful feature selection Limitations: Extensive preprocessing is required Lack of medical data sets Inability to generalize the results and trends for lack of comparison with other methods Validation: 10-fold cross validation repeated 10 times
[33]	Objective: Classification of chronic myeloid leukemia phases Data set: 500 pictures from Patliputra Medical College and Hospital, Dhanbad, and the blood journal repository Methodology: CNN	Performance: Accuracy: 97.8% Strengths: Use of transfer learning Limitations: Limited data set Validation: Internal validation (14 left for testing)

^a^CNN: convolutional neural network.

^b^SESSA: statistically enhanced salp swarm algorithm.

### Hematology Diagnosis

#### Machine Learning–Based Models

To address the challenge of manually detecting blasted cells, Dasariraju et al [54], Inbarani et al [66], Abedy et al [29], Jagadev and Virani [34], and Dharani and Hariprasath [31] used medical images of healthy and malignant samples to automatically identify the leukemic types and subtypes. While Dasariraju et al [54] applied an RF algorithm as an approach to differentiate between abnormal and healthy leukocytes, and classify immature leukocytes into their 4 subtypes, Inbarani et al [66] discussed the implementation of a novel sophisticated approach to identify ALL blast cells via the histogram-based soft covering rough K-means clustering (HSCRKM) segmentation algorithm. The latter is a hybrid-clustering technique that combines the strengths of both the soft covering rough set and the rough K-means clustering. Nevertheless, one main limitation of the HSCRKM segmentation technique is that it is not suitable for multiple color images because the latter increase the processing time due to an increase in the peak values of the histogram. To enhance image representation and prepare a clean input for the classification model, Jagadev and Virani [34] applied SVM on 220 blood smear images of healthy individuals and patients with leukemia to identify the 4 leukemic subtypes (AML, CML, CLL, and ALL) using both K-means clustering and hue, saturation, value color–based segmentation techniques. Similarly, Dasariraju et al [54] performed a set of morphological imaging modifications and preprocessing techniques to segment the nucleus and cytoplasm and overcome the difficulty of blood image detection. To reduce data dimensionality, the model’s speed, and processing time, the most relevant features were obtained using feature extraction techniques and the resulting output was passed on to the classifier [34,54,66]. Alternatively, Abedy et al [29] chose to employ the histogram of oriented gradients for feature extraction, the Gaussian filter for noise elimination, and the Sobel kernel for image filtering. Furthermore, Dey and Islam [49] adopted the principal component analysis technique followed by grid search for hyperparameter tuning, which significantly decreased the number of components from 7129 features to only 6 important parameters. This data transformation technique did not only reduce the computational time and made it faster, but it also helped giving better results. Alongside the usage of imaging techniques, many studies employed other techniques; for example, Moraes et al [133] suggested the usage of flow cytometry data for distinguishing leukemia/lymphoma, and Mahmood et al [143] directed their research to focus more on identifying the most discriminatory features for CLL using patient laboratory test results, demographic parameters, and training a Classification and Regression Trees model on 94 pediatric patients, which was evaluated using 10-fold cross validation. Moreover, both Dharani and Hariprasath [31] and Jagadev and Virani [34] used SVM to classify leukemia and its subtypes, while Paswan and Rathore [28] used K-nearest neighbors to separate blasted blood cells from normal ones and classify them further into either AML or ALL using a value of K=4. By contrast, Moraes et al [133] suggested the implementation of decision tree as an ML-based technique for distinguishing leukemia/lymphoma, where a binary classification between healthy and immature leukocytes was performed with an 80%/20% data split, followed by a subclassification of immature leukocytes into their respective 4 types using a 70%/30% split, and several combinations of hyperparameters were evaluated during a 5-fold cross validation. Conversely, Abedy et al [29] chose to employ logistic regression as a classifier to identify the shape of the leukemic cell from microscopic blood images, while Dey and Islam [49] conducted a study to detect patients’ leukemia type based on their gene expression information using the RF algorithm and 2 other algorithms, XGBoost and artificial neural networks (ANNs), and Dasariraju et al [54] used RF to perform a subclassification of immature leukocytes into their respective 4 types.

#### Deep Learning–Based Models

With the objective of optimizing leukemia diagnosis, some studies made use of genetic features. For example, Rodrigues and Deusdado [63] suggested the application of a kernel logistic regression that classifies gene expression data using meta-learners to select the most relevant attributes before classification. The data set used for training comprised the 2 types of leukemia (ALL and AML) and a set of gene features. Pearson correlation and chi-square statistic were the 2 approaches used on meta-learners to assess attributes. Then, all models used 10-fold cross validation resulting in an identification of 12 common genes. Other studies, such as that by Al-Dulaimi et al [23], focused on current practices, techniques, and challenges in digital hematology detection of WBCs and their components (nuclei and cytoplasm) using hematologic microscopy images. In addition to analyzing the growing trends in computer-aided diagnosis applications, the review highlighted the main challenges associated with the use of CNNs in terms of both high computational time and costs to classify images and detect abnormalities. This gave rise to the use of transfer learning and enhancing optimization techniques, such as the bare bones particle swarm optimization algorithm used by Srisukkham et al [12] to extract the most informative features and enhance the classification accuracy of the lymphocytic cells into either normal or blasted. Likewise, Miyoshi et al [122] directed their work toward enhancing lymphoma diagnosis by classifying histopathological lymphoma images using a DL model. The aim of the study was to evaluate the performance of the suggested automated DL model and compare it with that of a traditional manual hematopathology detection procedure using CNNs. In this study, each test set comprised a total of 100 image patches, and the rest was randomly divided into 5 separate groups. During each repetition for 5 iterations, 1 group was kept for validation to evaluate the classifier performance for every epoch, while the other 4 groups were used for training. The number of epochs used in this study was 30. Similarly, Shafique and Tehsin [125] suggested the deployment of deep CNNs to detect the ALL type and its corresponding subtypes, while Zhao et al [60] proposed turning the captured raw multiparameter flow cytometry data into a 2D image by means of a self-organizing map (SOM) to analyze and classify them using the aforementioned algorithms. An SOM is a map of neurons that relies on unsupervised learning, in the sense that human intervention is not necessarily required. This model is used in many applications and has strong generalization abilities. The model was trained for 15 epochs, at a learning rate of 0.001 using an Adam optimizer function. The evaluation of classification accuracies used a 10% validation split, which was also employed for network architecture optimization. Then, the model performance was evaluated using the hold-out test set. In the same context, Sipes and Li [4] attempted fine-grained image classification for ALL diagnosis and compared the accuracies of CNNs and other models that used specific hand-selected features, while Vincent et al [37] proposed a leukemia classification that could be performed on 2 levels. The first process was applied to well-segmented nuclei extracted from 100 blood smear images. In this step, 90 samples were used for training and 10 were kept for validation. The cells were classified into normal and abnormal. The second step consisted of feeding 5 features extracted from abnormal images into a second classifier that split the images into ALL and AML types accordingly. Table 6 summarizes some of the studies in the diagnosis phase based on their objectives, data sets used, methodologies, and performance characteristics.

**Table 6:** Study analysis for journal publications on the diagnosis phase.

Reference	Objective, data set, and methodology	Performance and remarks
[60]	Objective: Classification of mature B-cell neoplasm Data set: 20,622 routine diagnostic samples from Munich Leukemia Laboratory Methodology: CNN-SOM^a^ transformation	Performance: Accuracy: 95% Strengths: Large data set High accuracy Limitations: Nonuniform distribution of misclassifications due to similarity in flow cytometric profiles Validation: 10% validation split
[54]	Objective: Detection of immature leukocytes and their classification into 4 types Data set: Images extracted from a publicly available data set at The Cancer Imaging Archive Methodology: Random forest algorithm	Performance: Accuracy: 92.99% Strengths: High precision results for each class Limitations: High number of false positives leading to low precision and specificity Validation: 5-fold cross validation
[49]	Objective: Identification of the leukemia type based on patient genetic expression Data set: A sample of 7129 genes that represent the genetic expressions of 72 people from Kaggle Methodology: XGBoost, artificial neural networks, and random forest algorithm	Performance: Random forest accuracy: 80.8% XGBoost accuracy: 92.3% Strengths: Use of principal component analysis for dimensionality reduction and faster computation Use of grid search for the best hyperparameter selection Limitations: Small data set (72 people) Validation: Internal validation (65%/35% split)
[135]	Objective: Classification of lymphocytic cells Data set: The ALL-IDB2 Database Methodology: bare bones particle swarm optimization–based feature optimization	Performance: Accuracy: 94.94%-96.25% Strengths: A good performance on capturing prognostic chronic myeloid leukemia markers by the model Limitations: Challenge of capturing relationships between data types with no information loss in clinical clustering Validation: Validation on an external independent clinical trial
[34]	Objective: Detection of leukemia and its types Data set: 220 blood smear images from healthy individuals and patients with leukemia Methodology: support vector machine	Performance: Accuracy: Above 80% Strengths: Use of 3 segmentation methods Broader range of leukemia classification (types and subtypes) Limitations: Costly method based on imaging data Validation: Internal validation (train test split)
[122]	Objective: Automated detection of malignant lymphoma Data set: Prepared histopathologic images (388 sections, 259 diffuse large B-cell lymphomas, 89 follicular lymphomas, and 40 reactive lymphoid hyperplasia) Methodology: Deep neural network classifier	Performance: Accuracy: 97% Strengths: High accuracy outperforming 7 pathologists Model ensemble comprising 3 classifiers Limitations: Classifier requires a manual annotation Model not able to classify all the subtypes Validation: K-fold cross validation repeated 5 times
[37]	Objective: Multiclassification of leukemia Data set: 100 blood smear images Methodology: Neural network classifiers	Performance: Accuracy: 97.7% Strengths: Two-step neural network classifier Limitations: Limited data set (100 blood smear images) Validation: Internal validation (90 images used for training and 10 kept for validation)
[133]	Objective: Leukemia and lymphoma diagnosis Data set: 283 blood and bone marrow sample images from patients with leukemia and lymphoma Methodology: Decision tree	Performance: Correctness: 95% Strengths: Application of the LASSO algorithm for regularization Model robustness and strength against false negatives Limitations: Complexity of the decision tree and the risk of overfitting through the production of too large trees Validation: 30-fold cross validation
[66]	Objective: Leukemia image segmentation Data set: The Acute Lymphoblastic Leukemia Image Database Methodology: HSCRKM^b^/particle swarm optimization/K-means	Performance: Accuracy: 80% and above Strengths: Use of 7 machine learning methods Application of soft covering rough approximation Limitations: Suitable for medical images only Application on multiple color images increases the processing time Validation: Different train/test sizes were used for model evaluation
[144]	Objective: Determining the most predictive features for acute lymphoblastic leukemia identification Data set: 94 pediatric patient samples collected from the Department of Hematology and Oncology, Children Hospital and Institute of Child Health, Lahore Methodology: Random forest, boosting machine, C5.0 decision tree, and classification and regression trees	Performance: Accuracy: 87.4% Strengths: High accuracy Balanced data set Limitations: Small-scale study Few machine learning models Socioeconomic risk factors not selected automatically Validation: Internal validation (train/validation data) 10-fold cross validation
[31]	Objective: Leukemia diagnosis and its subtypes Data set: 200 blood smear images extracted from Vidyalankar Institute of Technology, Mumbai and online databases Methodology: support vector machine	Performance: Accuracy: 97.8% Strengths: Good detection accuracy Thorough image segmentation process Limitations: Challenging detection process due to the irregularity of the cancer cell’s shape and nucleus Use of only support vector machine for classification

^a^CNN-SOM: convolutional neural network-self-organizing map.

^b^HSCRKM: histogram-based soft covering rough K-means clustering.

### Hematology Treatment

#### Machine Learning–Based Models

Complete remission (CR) refers to the disappearance of all signs and symptoms of an illness [51]. However, a significant proportion of patients report disease relapse after therapy and complete disease recovery. In this regard, Gal et al [75] proposed an ML technique that uses gene expressions to predict the likelihood of CR in patients with AML who previously received therapy. The 473 collected samples were divided into training and testing and were fed into the 3 classifiers for feature selection using a 5-fold cross validation. To select the most significant genes that clearly mark the difference between the state of CR and non-CR, a statistical *t* test was performed in each fold for each method. For further gene feature selection and performance enhancement, the results were compared with those of 3 algorithms: randomized LASSO, recursive feature elimination, and hill climbing. It was proven that cancers that appear pathologically identical do not necessarily exhibit the exact response to similar drugs. To overcome the challenge of high demands for personalized or patient-specific medicine, Lee et al [9] proposed using a gene-expression profile and in vitro drug sensitivity data to spot molecular markers that explain this patient-specific drug response. The data set used comprised 160 chemotherapy drugs and inhibitors for 30 patients with AML. The gene-drug association was identified using the MERGE algorithm, which utilized gene characteristics such as a novel mutation, expression hubness, known regulator, genomic copy number variation, and methylation. The model testing was done in 2 different ways: the first approach used 2 batches containing 12 patient samples for training and 12 different samples for validation, respectively. The second approach used a leave-one-out cross validation to test the predicted drug sensitivity for 30 patient samples. The latter cross-validation method is regarded as reasonable because it prevents the high computational training cost and time owing to the small sample size and is much less biased than using a single test set because the process fits the data set consisting of n-1 observations repeatedly [145]. Although having a limited data size permitted the obtention of efficient validation results, identifying gene-drug associations is deemed to be a challenging process in that case. However, the proposed algorithm was successful to prioritize genes based on the multidimensional data on their potential to drive cancer. Consequently, upon comparison with other alternate methods (ElasticNet, multitask learning, Pearson P value, and Spearman P value), MERGE exhibited the best gene-drug association result.

#### Deep Learning–Based Models

In their review and analysis of current AI applications for hematologic disorders’ treatment, Muhsen et al [24] presented a study that was performed with a set of patients who underwent allogeneic hematopoietic cell transplantation to predict the development of acute graft-versus-host disease using ANNs. After comparing the performance acquired by ANNs and the results achieved by logistic regression, ANNs were found to predict the presence of graft-versus-host disease significantly better. However, among the limitations remaining to help reach optimal ML results is the limited data input from patients that could be further enlarged to include biologic and genetic factors, for instance. Moreover, the employment of several sampling techniques such as random oversampling, synthetic oversampling, and remote under sampling could help improve the ML models’ accuracies in predicting treatment-related mortality in allogeneic hematopoietic cell transplantation.

Similarly, Lyu et al [42] used ANNs to classify the progress and change in gene expressions and peripheral blood mononuclear cells before treatment and after starting therapy. Table 7 shows some extracted literature focusing on the hematology treatment phase.

**Table 7:** Study analysis for journal publications on the treatment phase.

Reference	Objective, data set, and methodology	Performance and remarks
[9]	Objective: Digital analysis of blood smears and preclassification of cells Data set: Images of blood smears from a hematologic laboratory Methodology: MERGE algorithm	Performance: Accuracy: 90% Strengths: Introduction of a new computational and statistical method to determine gene markers Limitations: Small data set comprising only 30 patients with acute myeloid leukemia Validation: Leave-one-out cross validation
[75]	Objective: Prediction of complete remission of acute myeloid leukemia Data set: 473 bone marrow samples from the Children’s Oncology Group Methodology: K-nearest neighbor, support vector machine, and hill climbing	Performance: Area under the curve: 0.84 Strengths: Use of 3 feature selection algorithms: randomized LASSO, recursive feature elimination, and hill climbing Use of 3 classifiers: support vector machine, random forest, and K-nearest neighbor Limitations: Small data set Validation: 100 iterations of a 5-fold cross validation
[81]	Objective: Identify the right patterns to improve risk stratification of patient with CLLs^a^ Data set: (1) the first cohort comprised CLL cells of 196 individuals; the second cohort comprised CLL cells of 98 individuals including their clinical data and RNA-seq Methodology: (1) EM algorithm and the Gaussian mixture models; (2) Boosted tree ensemble method	Performance: Precision: 90% Strengths: High accuracy and precision Limitations: Large data set and 5-year monitoring is required Validation: External validation on an independent cohort

^a^CLL: chronic lymphocytic leukemia.

### Conclusions and Future Research Directions

Early diagnosis and prediction of hematologic malignancies can immensely reduce mortality rates and can improve patient survival rates. Nevertheless, the nature of data on medical treatment is complex and requires an in-depth analysis to extract the important explicative features and hidden data patterns. The only way to handle enormous sets is through the use of AI. The challenge that faces AI applications, however, is the limitation in data availability, which can be overcome by means of data augmentation techniques, regularization, and transfer learning. This review of the literature highlights the most recent applications of both DL and ML in the field of blood cancer management for every hematologic pathway stage and malignancy type. Based on the reviewed articles, ML techniques have been widely used, in comparison with DL methods, as the latter are relatively newer and require larger data sets than ML, which is considered a constraint in the medical field. In some studies, ML techniques performed better than DL methods and vice versa, depending on the application and nature of data used. Moreover, screening and diagnosis are challenging tasks, as hematologic cancers are difficult to identify during their initial stages. Therefore, many studies in the field investigated the aforementioned stages alongside prediction, while less attention has been paid to the treatment stage. The latter is critical and requires further analysis and study, as repercussions and relapses may arise due to cancer treatment, namely, chemotherapy, which requires a risk evaluation and future mitigation plans. Furthermore, some malignancies appeared to be more addressed than others, mainly acute myeloid/lymphoblastic leukemias that have gotten the most attention in the last few years followed by lymphoma, due to their fast development and aggressivity. Conversely, there was less emphasis and minor existing literature tackling the chronic types of leukemia. This can be due to the slow-growing pattern of the aforementioned, and a lack of sudden symptom exhibition until very late stages, which makes the latter’s monitoring quite complex. Overall, a lack of detection accuracy can have a significant impact on the patient’s journey to treatment because it can delay diagnosis and affect the efficiency of therapies. Therefore, predictive models that can recognize disease patterns and common symptoms in hematologic malignancies based on medical patient records are essential to forecast the risk of infection and avoid late-stage diagnoses. Thus far, many studies employed several techniques to predict hematologic malignancies’ diagnosis through either medical image recognition, flow cytometry, or genetic expressions. However, no study in the literature has ever made use of patient CBC test results alone for blood disorder prediction or detection purposes. As the latter is generally regarded as the first diagnostic routine for hematologists to confirm leukemia diagnosis, it can be an efficient medium to potentially investigate in the future.

## Abbreviations

AI: artificial intelligence
ALL: acute lymphoblastic leukemia
AML: acute myeloid leukemia
ANN: artificial neural network
AUC: area under the curve
CBC: complete blood count
CLL: chronic lymphocytic leukemia
CLL-TIM: CLL Treatment-Infection Model
CML: chronic myeloid leukemia
CNN: convolutional neural network
CR: complete remission
DL: deep learning
DNN: deep neural network
GOTDP-MP-CNNs: globally optimized transfer deep-learning platform with multiple pretrained CNNs
HSCRKM: histogram-based soft covering rough K-means clustering
IPI: International Prognostic Index
ML: machine learning
RBC: red blood cell
RF: random forest
ROC: receiver operating characteristic
SEER: Surveillance, Epidemiology, and End Results
SESSA: statistically enhanced Salp Swarm Algorithm
SOM: self-organizing map
SVM: support vector machine
WBC: white blood cell
